# RU AGE-FRIENDLY? THE RUTGERS AGING INITIATIVE

**DOI:** 10.1093/geroni/igad104.3580

**Published:** 2023-12-21

**Authors:** Lauren Snedeker, Tracy Davis

**Affiliations:** Rutgers School of Social Work, New Brunswick, New Jersey, United States; School of Health Professions, Rutgers, Rutgers, New Jersey, United States

## Abstract

Ageism is harmful to everyone everywhere, including university settings. Rutgers is among America’s highest-ranked, most diverse public research universities. While efforts have been made to increase diversity, equity, and inclusion, few efforts have focused specifically on ageism. Like many individuals and groups experience, ageism is often the last experience identified in the DEI equation (can cite if needed). The objective of this project was to begin to take steps to end ageism at Rutgers University and beyond and move towards becoming an age-friendly university. As a part of this project, we surveyed 109 full or part time faculty members about their understanding of ageism and their experiences with ageism at the university. Furthermore, faculty were asked about the feasibility of including ageism into their courses and whether they would need training or materials to do so. Our survey results indicate that faculty feel prepared to lead discussions on ageism with students, recognize its importance in advancing equity and inclusion, and but largely lack any formalized training on the matter. Our project, supported by a university grant, helped respond to these findings and create a sustainable action plan for the future that ensures ageism and age inclusivity are experiences that continue to be addressed at the university level.

